# Grazing rest during spring regreening period promotes the ecological restoration of degraded alpine meadow vegetation through enhanced plant photosynthesis and respiration

**DOI:** 10.3389/fpls.2022.1008550

**Published:** 2022-10-03

**Authors:** Ying Liu

**Affiliations:** Qinghai Academy of Animal Husbandry and Veterinary Sciences, Qinghai Provincial Key Laboratory of Adaptive Management on Alpine Grassland, Key Laboratory of Superior Forage Germplasm in the Qinghai–Tibetan Plateau, Qinghai University, Xining, China

**Keywords:** photosynthesis, Calvin cycle, respiration, TCA cycle, grazing rest during spring regreening period

## Abstract

Grazing rest during the spring regreening period is the most economical and feasible measure for the ecological restoration of degraded alpine meadows and has been widely popularized and applied in China. The aim of the present study was to undertake a comparative analysis of the effects of grazing rest on the ecological restoration of degraded alpine meadows by plant photosynthesis and respiration. Coverage, height, ground biomass, belowground biomass of degraded alpine meadow vegetation, net photosynthetic rate, stomatal conductance, transpiration rate, intercellular CO_2_ concentration, chlorophyll fluorescence parameters, relative chlorophyll content, respiration rate, metabolite content, leaf relative water content, and related mineral element content of the dominant grass *Elymus nutans* Griseb. were measured in degraded alpine grassland with different grazing rest years. The results show that grazing rest during the spring regreening period promoted the ecological restoration of degraded alpine meadows by enhancing the photosynthesis and respiration of the dominant grass *E. nutans* Griseb. Grazing rest enhanced photosynthesis in dominant grass by increasing metabolites related to the Calvin cycle, chlorophyll content, leaf relative water content, and related mineral element content. Grazing at rest enhanced the respiration of dominant grass by increasing metabolites related to the TCA cycle, leaf relative water content, and related mineral element content. This positive effect gradually became stable with increasing years of grazing rest. Our results provide a fundamental basis for the popularization and application of grazing rest during the spring regreening period on degraded Tibetan Plateau grasslands.

## Introduction

The Qinghai–Tibet Plateau is a sensitive and ecologically fragile zone of global climate change. Because of global climate change and human activities, alpine grasslands on the Qinghai–Tibet Plateau continue to degrade, and the structure and function of the ecosystem are seriously disturbed (Fan et al., 2010). Overgrazing is the main reason for the degradation of alpine grasslands (Zimmer et al., 2010; Ash et al., 2011; Selemani et al., 2013; Li et al., 2016; Shang et al., 2017); therefore, short-term grazing rest has become an effective way to control degraded grasslands and perform natural restoration (Wu et al., 2017). The forage spring regreening period refers to the stage at which plants end their dormant state and begin to recover with an increase in temperature and moisture conditions after forage overwintering. This is the most important stage for the initial growth of grassland vegetation in a year (Zhang et al., 2011). Consequently, the implementation of grazing rest during the spring regreening period is an effective approach for the natural restoration and rational utilization of degraded grasslands. How grazing rest during the spring regreening period promotes the restoration of degraded grasslands remains a research hotspot (Mavromihalis et al., 2013; Li et al., 2017; Fedrigo et al., 2018). The answer to this scientific question can not only fill in the mechanism of the significant restoration of degraded grassland by grazing rest during spring regreening period but also provide a strong theoretical basis for the promotion of grazing rest during spring regreening period measures and further provide guidance for grassland management policies in China.

Carbon assimilation and utilization by plants play an important role in the restoration of degraded alpine grassland vegetation, and the main processes of carbon metabolism are photosynthesis and respiration (Liu et al., 2008; Chamizo et al., 2021). Photosynthesis and respiration are also sensitive processes in response to environmental changes (Martínkov et al., 2021; Crous et al., 2022). These can directly reflect the growth status of grassland plants (Chen et al., 2005). Zhao et al. (2009) and Zheng et al. (2011) reported that overgrazing significantly reduces the photosynthetic and transpiration rates of forage plants, and chlorophyll fluorescence parameters are appropriate for detecting the effect of environmental factors (Simkó et al., 2020). Chlorophyll is essential for plant photosynthesis. Livestock grazing can affect the chlorophyll content in steppe plants of Tuva (Zvereva, 2004). Plant respiration refers to the process by which plants absorb O_2_ or release CO_2_ per unit time, which provides most of the energy required for plant life activities (Noguchi and Yoshida, 2008). Shen et al. (2013) reported that grazing affects the respiration rate of grassland plants. In addition, photosynthesis and respiration are inseparable from water and mineral elements—for example, nitrogen (N), phosphorus (P), and potassium (K) are essential elements in photosynthesis and respiration (Brooks, 1986; Nobuyuki et al., 2008; Zhang et al., 2017). Magnesium (Mg)-containing chelatase is the first enzyme in the chlorophyll biosynthetic pathway (Rissler et al., 2002). Copper (Cu) is vital for photosynthetic and respiratory electron transport processes and other cellular redox reactions (Biswas et al., 2013), and manganese (Mn) is an essential component of chloroplasts (Anja and Sébastien, 2018). However, there is a dearth of information regarding the reasons underlying this grazing rest-induced effect on forage photosynthesis and respiration. Metabolomics is an emerging approach in the post-genome era, which can comprehensively analyze the changes in metabolite content in plants and their dynamic responses to exogenous environmental factors (Ning et al., 2013). Therefore, this approach is a good choice to explore the response mechanism of photosynthesis and respiration processes to grazing rest.

The objectives of this study were (a) to identify the effects on the physiological characteristics of degraded alpine meadow vegetation after varying years of grazing rest during the spring regreening period and (b) to determine how priming of forage with grazing rest during the spring regreening period affects photosynthesis and respiration in a dominant grass (*E. nutans* Griseb.) for the degraded alpine meadow vegetation.

## Materials and methods

### Plant materials and treatments

The study site is located in Wariga Village, Mole Town, Qilian County, Qinghai Province, with a geographical location of 37°56′ N, 100°13′ E and an altitude of 3,650 m mainly containing alpine meadow soil. The grassland is a typical alpine meadow vegetation. The main species were *Kobresia pygmaea*, *Kobresia humilis*, *E. nutans* Griseb., and *Poa crymophila*. A relatively uniform natural alpine meadow was selected as the test area, with 24 hm^2^ as the treatment area and the other 6 hm^2^ as the control. The grassland utilization patterns were winter and spring pastures. The grassland degradation level in the experimental area was moderate (*i*.*e*., the proportion of edible grass in the grassland was 5–15%). The mean value of the treatment area was divided into four parts. Grazing rest was implemented during the green-up period from 2015, 2016, 2017, and 2018 (the green-up period was from May 10 to July 10): treatment 1, grazing rest during the green-up period for 1 year (2018); treatment 2, grazing rest during the green-up period for 2 years (2017 and 2018); treatment 3, grazing rest during the green-up period for 3 years (2016, 2017, and 2018); and treatment 4, grazing rest during the green-up period for 4 years (2015, 2016, 2017, and 2018). The control area was free grazing according to local traditional (with moderately severe grazing intensity and utilization rate of forage grass above 50%). The control and treatment areas were three replicates, each with 2 hm^2^. The total grassland coverage, ground biomass, belowground biomass and height, photosynthetic characteristics, chlorophyll fluorescence parameters, respiration rate, relative chlorophyll content, and leaf relative water content of the dominant species’ leaves were measured at nine representative points in each replicate, and the indices were measured in August 2018. Based on the preliminary test results of our research group, the dominant species selected in this study was *E. nutans* Griseb.

### Quantitative characteristics of alpine meadow

A quadrate method was used to determine the quantitative characteristics of grassland vegetation, and the quadrate area was 50 cm × 50 cm. The specific method was as follows: the total coverage of vegetation in the quadrate was evaluated by visual measurement. A steel tape was used to select three plants of *E. nutans* Griseb. in each square to measure the natural plant height, which was calculated as the average plant height of the dominant herbage species in the quadrate. All plants in the quadrate square on the ground were cut, put in an envelope bag, and brought to the laboratory to be dried to constant weight at 75°C. Their dry weight was the ground biomass. Five samples (0–15 cm) of the plant underground root system were taken from the quadrate with a root drill (inner diameter: 5 cm) and dried to constant weight after being washed with clean water, which was converted into the belowground biomass of vegetation in the quadrate.

### Leaf water and chlorophyll contents

Ten representative *E. nutans* plants were selected, of which the leaves were cut and weighed as fresh weight and then brought to the laboratory for drying to a constant weight at 75°C. The dry weight was obtained, and the leaf water content was calculated. Relative chlorophyll content was measured using a chlorophyll meter (SPAD-502).

### Photosynthesis and respiration parameters

The photosynthetic characteristics, chlorophyll fluorescence parameters, and respiration rate of *Elymus nutans* were measured using li-COR 6400XT (LI-COR, Lincoln, NE). The photosynthetic characteristics included the net photosynthetic rate (Pn), transpiration rate (Tr), intercellular CO_2_ concentration (Ci), stomatal conductance (Gs), respiration rate (R), and fluorescence parameters. The chlorophyll fluorescence parameters included photochemical quantum efficiency (Fv/Fm), (Fv′/Fm′), actual photochemical quantum efficiency (φPSII), photochemical quenching coefficient (qP), and electron transfer rate (ETR). After being induced by natural light for 1.5–2 h, an open air path was adopted. According to the average temperature of 09:00–12:00 during the measurement period, the temperature of the measuring chamber (T-block) was set to 25°C, and the flow rate was 500 mol S^-1^. After the gas exchange parameters were measured, the light source was closed. The leaves were maintained in the dark for 30 min for adaptation before measuring the minimum (F0) and maximum (Fm) fluorescence.

### Metabolite content analysis

Representative and healthy two-leaf pots from each treatment replicate were selected as the six replicates for metabolite content analysis. Fresh leaves (0.1 g) were collected from each replicate (pot) for each treatment, immediately frozen in liquid nitrogen, and stored at −80°C for subsequent analysis. The extraction protocol used was modified from that described by Du et al. (2012). The frozen leaves were ground to a fine powder with liquid N_2_, and then 100 mg of the powdered leaves was weighed in a 2-ml centrifuge tube. Then, 750 μl of methanol, 250 μl of chloroform, and 100 μl of aqueous chlorphenylalanine solution (3 mg ml^–1^, as the internal standard solution) were added to the tube, and the solution was extracted at 60 Hz for 5 min in an ultrasonic water bath. The extraction solution was centrifuged at 12,000 × *g* for 10 min at 4°C, and then 400 μl of the polar phase was decanted and dried in a Centrivap benchtop centrifugal vacuum concentrator (Labconco, Kansas City, MI, USA). The dried polar phase was incubated for 90 min at 37°C with 100 μl methoxyamine hydrochloride (20 mg ml^–1^) in pyridine and then incubated with 100 μl bis (trimethylsilyl) trifluoroacetamide for 1 h at 70°C. After methoximation and trimethylsilylation, the extracts were analyzed according to Du et al. (2013) using a gas chromatograph–mass spectrometer (TurboMass-Autosystem XL; PerkinElmer, Waltham, MA, USA). The metabolites detected were identified using the Turbomass 4.1.1 software (PerkinElmer) coupled with commercially available compound libraries: NIST 2005 (PerkinElmer, Waltham, MS) and Wiley 7.0 (John Wiley & Sons, Hoboken, NJ).

### Mineral element content analysis

Fresh and healthy leaves were dried at 60°C until a constant weight was achieved. The dried leaves were ground, and 1 g of powdered sample was weighed. Then, the weighed particulates were digested with a mixture of H_2_SO_4_ and H_2_O_2_ for further N and P determination. Total N was analyzed using a Kjeltec 2300 analyzer (Foss Tecator AB, Hoeganaes, Sweden), and the vanadium molybdate yellow colorimetric method was used to determine the total leaf P content.

The powdered samples (1 g) were placed in a silica crucible for K, Mg, Cu, and Mn determination (25 ml). The silica crucible was heated at 550°C for 3 h, and 2 ml of double-distilled water was added when the silica crucible was cooled. Then, 10 ml of 6.00 mol/L muriatic acid was added to the silica crucible at 25°C and was heated again until dry. Subsequently, 5 ml of 6.00 mol L^–1^ muriatic acid was added to the silica crucible and then dissolved in double-distilled water up to 50.00 ml for further determination of K, Mg, Cu, and Mn. The blank control group was subjected to the same procedure. K, Mg, Cu, and Mn were determined using an atomic absorption spectrophotometer (SOLAAR, Thermo Elemental) at 766.5, 285.2, 324.8, and 279.5 nm, respectively. The detection limits (micrograms per milliliter) of the four elements were 0.2474, 0.1650, 0.0633 and 0.0306. The instruments were calibrated using standard solutions (0.20–100 μg ml^–1^) for the above-mentioned elements.

### Statistical analysis

Data were preliminarily sorted and statistically analyzed using Excel 2010, and an independent sample *t*-test was conducted using SPSS 20.0, with a significance level of 0.05. Plot analysis was performed using Sigma Plot 12.5, and correlation analysis was conducted using R language.

## Results

### Effects of grazing rest during the spring regreening period on the quantitative characteristics of degraded alpine meadow vegetation

**Figure 1:**
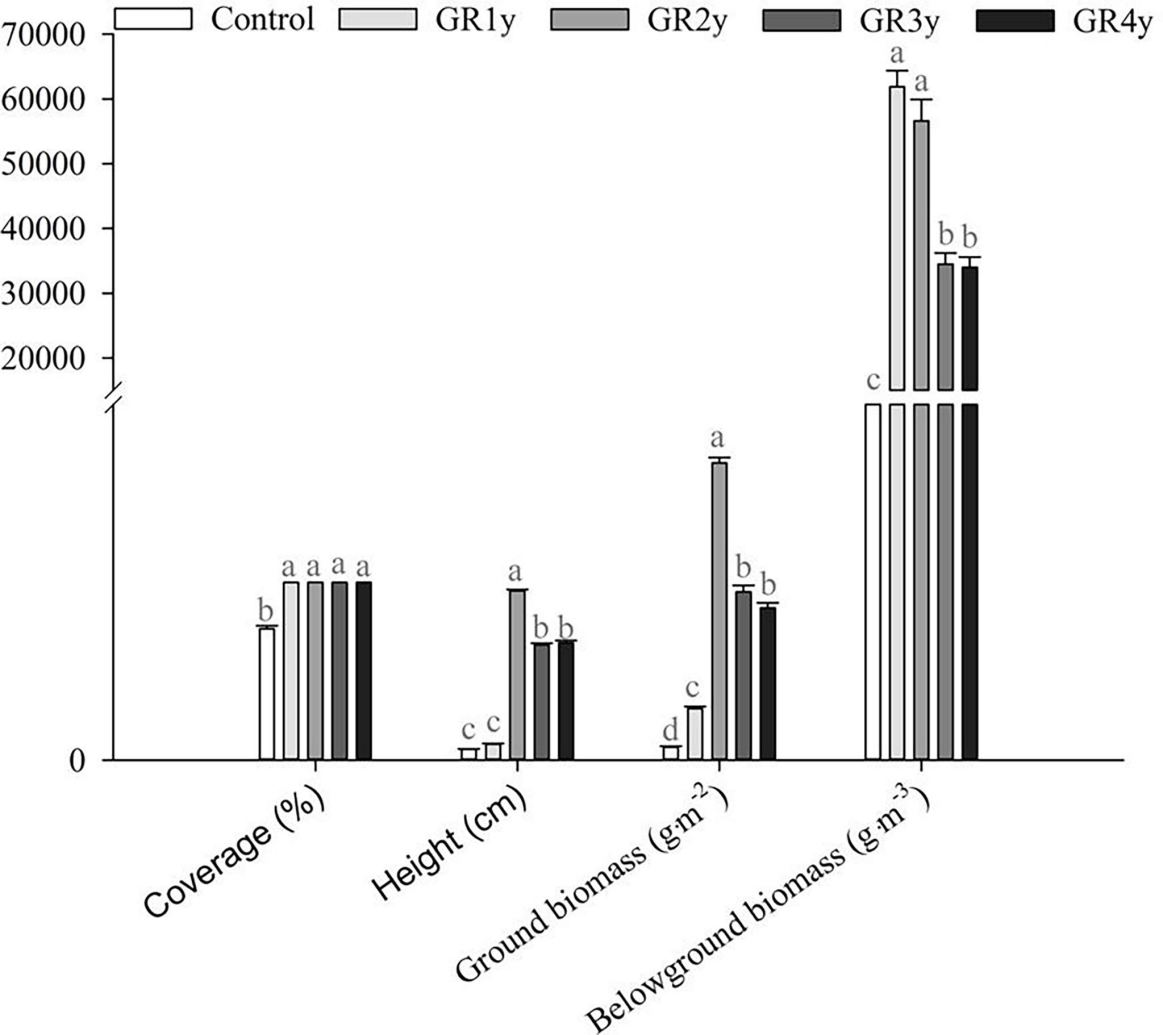
Effect of grazing rest during the spring regreening period on the quantity characteristics of the degraded alpine meadow vegetation. Bars represent SEs. Common letters above columns indicate no significant difference at *P* = .05. GR 1y, GR 2y, GR 3y, and GR 4y mean grazing rest for 1, 2, 3, and 4 years, respectively.

The quantitative characteristics of degraded alpine meadow vegetation included coverage, height, ground biomass, and belowground biomass. In this study, grazing rest during the spring regreening period significantly increased the coverage, height, ground biomass, and belowground biomass of degraded alpine meadow vegetation compared with the control (**Figure 1**). The coverage significantly increased from grazing rest during the spring regreening period of 1 year. There was no significant difference in coverage between grazing rests for one to four years. The height was significantly increased from grazing rest during the spring regreening period for 2 years, but then it decreased for grazing rest for 3 or 4 years. There was no significant difference in height between grazing rests for 3 and 4 years. The ground biomass significantly increased from grazing rest during the spring regreening period for 1 year and then approached the peak level for grazing for the remaining 2 years. The ground biomass did not differ significantly between grazing rests for 3 and 4 years. The belowground biomass significantly increased from grazing rest during the spring regreening period for 1 year but then decreased for grazing rest for 3 or 4 years. The belowground biomass did not differ significantly between grazing rests for 3 and 4 years.

### Effects of grazing rest during the spring regreening period on the photosynthesis of degraded alpine meadow vegetation’s dominant grass *E. nutans* Griseb.

**Figure 2:**
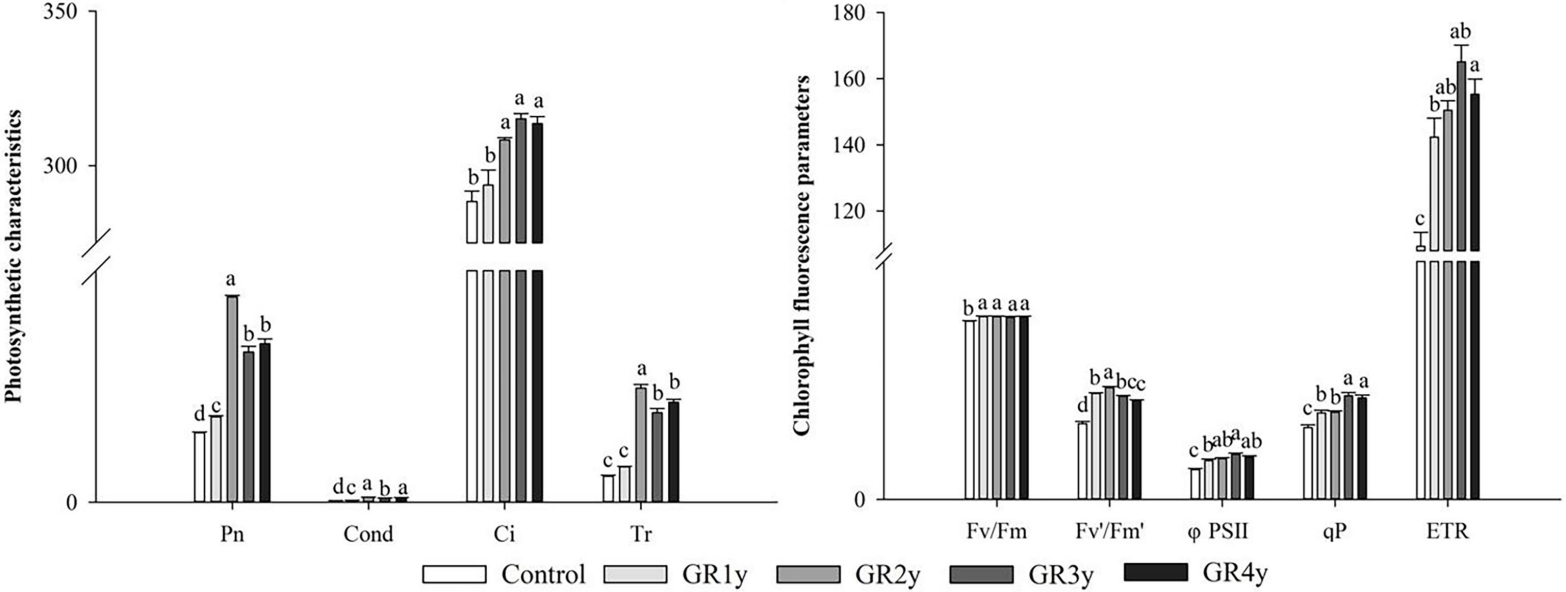
Effect of grazing rest during the spring regreening period on the photosynthesis of the dominant grass *Elymus nutans* Griseb of the degraded alpine meadow vegetation. Bars represent SEs. Common letters above columns indicate no significant difference at *P* = .05. GR 1y, GR 2y, GR 3y, and GR 4y mean grazing rest for 1, 2, 3, and 4 years, respectively.

All photosynthetic characteristics were significantly increased by grazing rest during the spring regreening period for several years compared with the control (**Figure 2**). The Pn, Cond, and Tr approached peak levels for grazing for the remaining 2 years. The Ci significantly increased for grazing rest during the spring regreening period of 2, 3, or 4 years, and there was no significant difference between them.

All chlorophyll fluorescence parameters were significantly increased by grazing rest during the spring regreening period for several years compared with the control (**Figure 2**). The maximal quantum yield of PSII (Fv/Fm) was not significantly different between grazing rests for 1, 2, 3, or 4 years. The photochemical efficiency of PSII in the light (Fv′/Fm′), actual photochemical quantum efficiency (φPS II), and ETR tended to increase first and then decrease. The photochemical quenching coefficient (qP) approached a peak level for grazing rest of 3 or 4 years.

**Figure 3:**
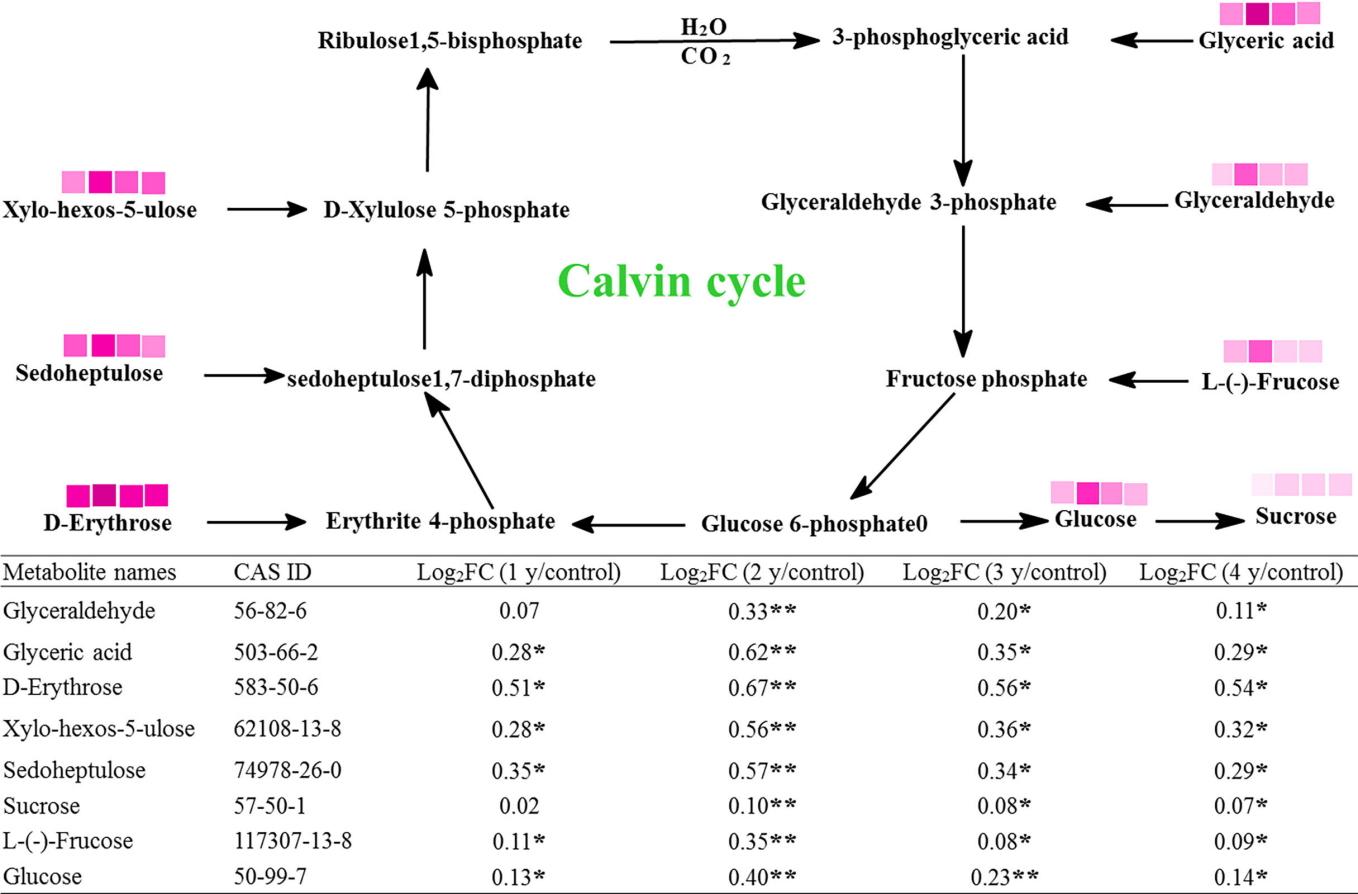
Effect of grazing rest during the spring regreening period on the Calvin cycle of the dominant grass *Elymus nutans* Griseb of the degraded alpine meadow vegetation. FC, fold change of relative content; *significant difference at *P* = .05; **significant difference at *P* = .01 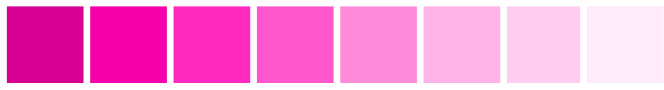 represent log_2_FC ≤0.7, ≤0.6, ≤0.5, ≤0.4, ≤0.3, ≤0.2, ≤0.1, and ≤0.05, respectively.

The Calvin cycle is an important photosynthetic process. In this study, there were eight metabolites associated with this cycle, which were significantly changed by grazing rest during the spring regreening period for several years (**Figure 3**). The contents of all eight metabolites were significantly increased by grazing rest during the spring regreening period for 2, 3, and 4 years compared with the control, and the value of log_2_FC reached a maximum at grazing rest for 2 years.

### Effects of grazing rest during the spring regreening period on the chlorophyll synthesis of degraded alpine meadow vegetation’s dominant grass *E. nutans* Griseb.

**Figure 4:**
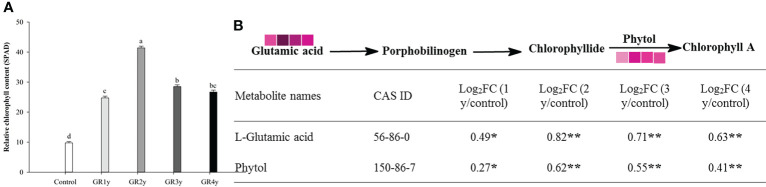
Effect of grazing rest during the spring regreening period on chlorophyll synthesis of degraded alpine meadows vegetation dominant grass Elymus nutans Griseb. **(A)** Effect of grazing rest during the spring regreening period on relative chlorophyll content of *Elymus nutans* Griseb. **(B)** Effect of grazing rest during the spring regreening period on metabolite contents in chlorophyll synthesis pathway of Elymus nutans Griseb. Bars represent SEs. Common letters above columns indicate no significant difference at *P* = .05. FC means fold change of relative content. * indicate significant difference at *P* = .05. ** indicate significant difference at *P* = .01. GR 1y, GR 2y, GR 3y and GR 4y mean grazing rest for 1 year, 2 years, 3 years and 4 years respectively 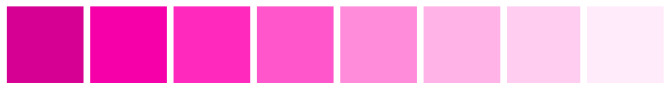
represent log_2_FC ≤0.7, ≤0.6, ≤0.5, ≤0.4, ≤0.3, ≤0.2, ≤0.1, and ≤0.05, respectively.

Grazing rest during the spring regreening period significantly increased the relative chlorophyll content for several years compared with the control (**Figure 4A**). The relative chlorophyll content approached the peak level for grazing for the remaining 2 years, and there was no significant difference between grazing rest for 3 or 4 years. There were two metabolites associated with the chlorophyll synthesis pathway, which were significantly changed by grazing rest during the spring regreening period (**Figure 4B**). The contents of the two metabolites were significantly increased by grazing rest for 1, 2, 3, and 4 years compared with the control, and the value of log_2_FC reached a maximum after 2 years of grazing rest.

### Effects of grazing rest during the spring regreening period on the respiration of degraded alpine meadow vegetation’s dominant grass *E. nutans* Griseb.

**Figure 5:**
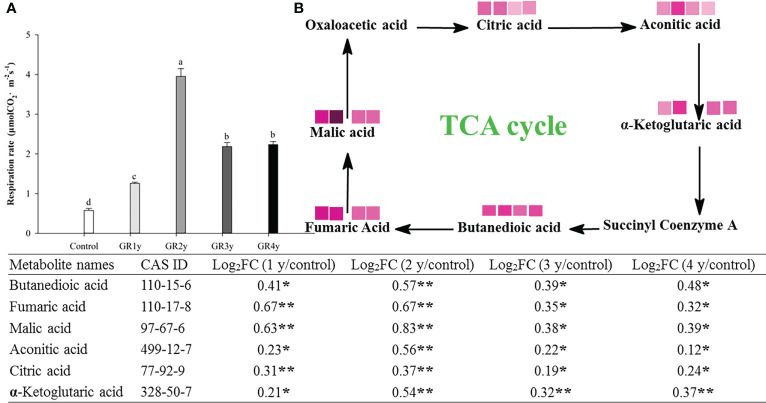
Effect of grazing rest during the spring regreening period on respiration of degraded alpine meadows vegetation dominant grass Elymus nutans Griseb. **(A)** Effect of grazing rest during the spring regreening period on respiration rate of *Elymus nutans* Griseb. **(B)** Effect of grazing rest during the spring regreening period on metabolite contents in TCA Cycle of Elymus nutans Griseb. Bars represent SEs. Common letters above columns indicate no significant difference at *P* = .05. FC means fold change of relative content. * indicate significant difference at *P* = .05. ** indicate significant difference at *P* = .01. GR 1y, GR 2y, GR 3y and GR 4y mean grazing rest for 1 year, 2 years, 3 years and 4 years respectively. represent Log2FC ≤0.7, ≤0.6, ≤0.5, ≤0.4, ≤0.3, ≤0.2, ≤0.1 and ≤0.05 respectively. 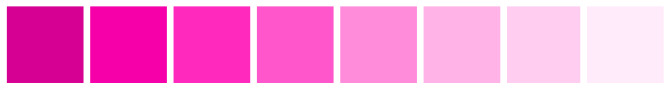
represent log2FC ≤0.7, ≤0.6, ≤0.5, ≤0.4, ≤0.3, ≤0.2, ≤0.1, and ≤0.05, respectively.

The respiration rate first increased gradually to the highest levels and then reduced owing to grazing rest during the spring regreening period, with the peak at grazing rest for 2 years. The TCA cycle is a critical link in the entire respiration process (**Figure 5A**). We found six metabolites, the contents of which significantly increased in this cycle caused by grazing rest during the spring regreening period for several years compared with the control (**Figure 5B**). The value of log_2_FC decreased after the peak at grazing rest for 2 years, and there was no significant difference between grazing rest for 3 and 4 years.

### Effects of grazing rest during the spring regreening period on the leaf water and mineral contents of degraded alpine meadow vegetation’s dominant grass *E. nutans* Griseb.

**Figure 6:**
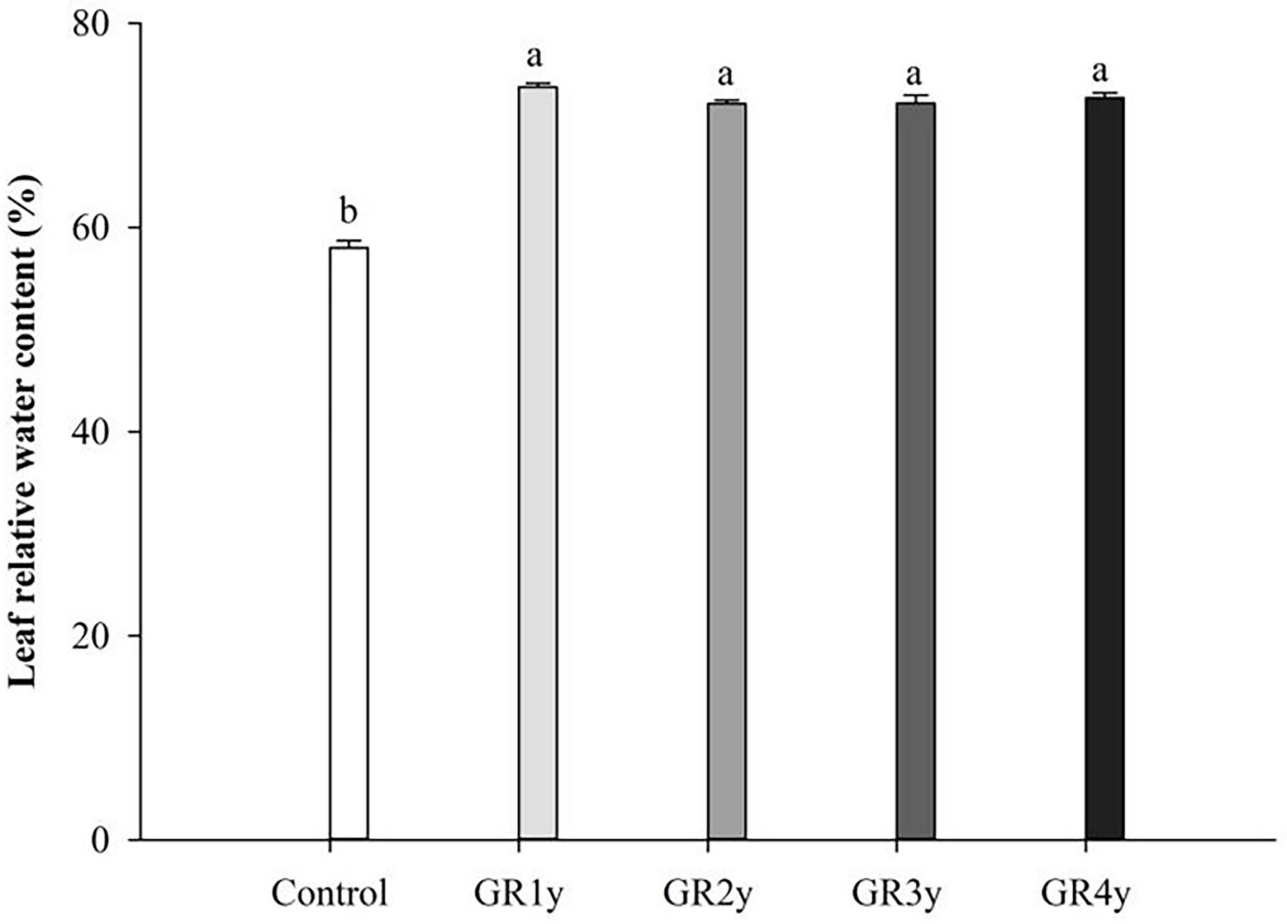
Effect of grazing rest during the spring regreening period on the leaf water content of the degraded alpine meadow vegetation. Bars represent SEs. Common letters above columns indicate no significant difference at *P* = .05. GR 1y, GR 2y, GR 3y, and GR 4y mean grazing rest for 1, 2, 3, and 4 years, respectively.

**Figure 7:**
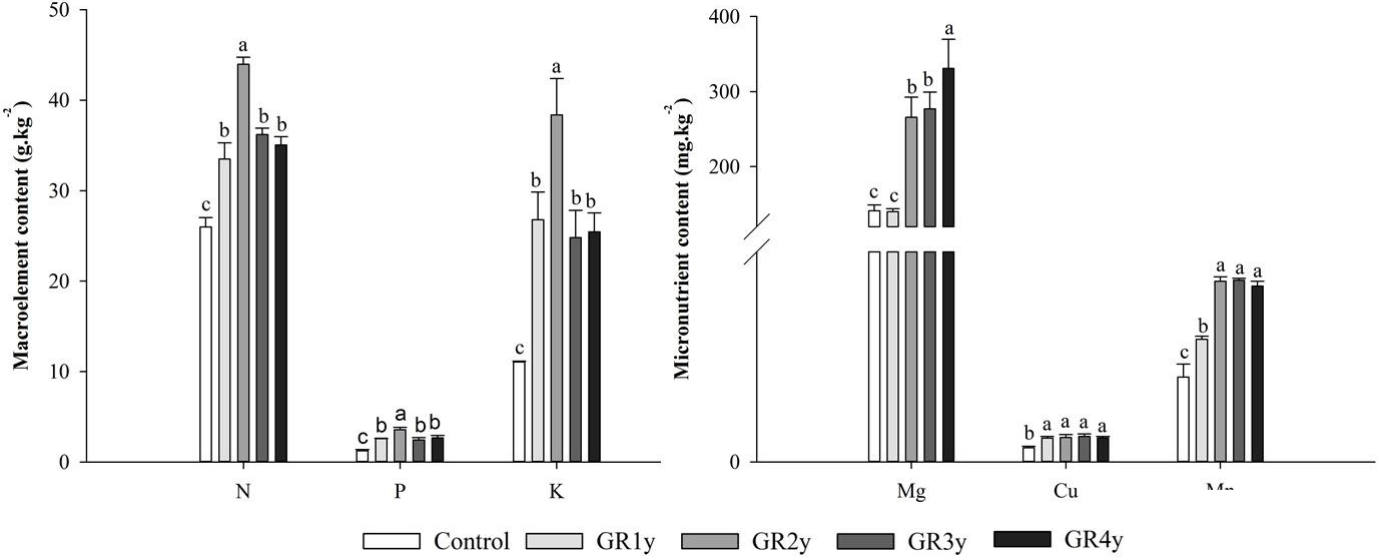
Effect of grazing rest during the spring regreening period on the mineral element content of the degraded alpine meadow vegetation. Bars represent SEs. Common letters above columns indicate no significant difference at *P* = .05. GR 1y, GR 2y, GR 3y, and GR 4y mean grazing rest for 1, 2, 3, and 4 years, respectively.

Grazing rest during the spring regreening period significantly increased the leaf relative water content in all rest periods. The leaf water content was not significantly different between grazing rests for 1 to 4 years (**Figure 6**). The macro elements (N, P, and K) showed the same variation tendency that increased first and approached the peak at grazing rest for 2 years and then decreased. There was no significant difference between grazing rest for 3 and 4 years. Mg showed an increasing trend year by year and was significantly increased by grazing rest for 2, 3, and 4 years. The Cu content of grass leaves significantly increased by grazing rest during the spring regreening period, and there was no difference between the years of grazing rest. Grazing rest during the spring regreening period significantly increased the Mn content of the leaves. The Mn content under grazing rest for 2, 3, and 4 years was more than 1 year, and under the latter 3 years, the content tended to be stable (**Figure 7**).

### Correlation analysis of vegetation quantitative characteristics, photosynthetic characteristics, respiratory characteristics, and key influencing factors of degraded alpine meadows

**Figure 8:**
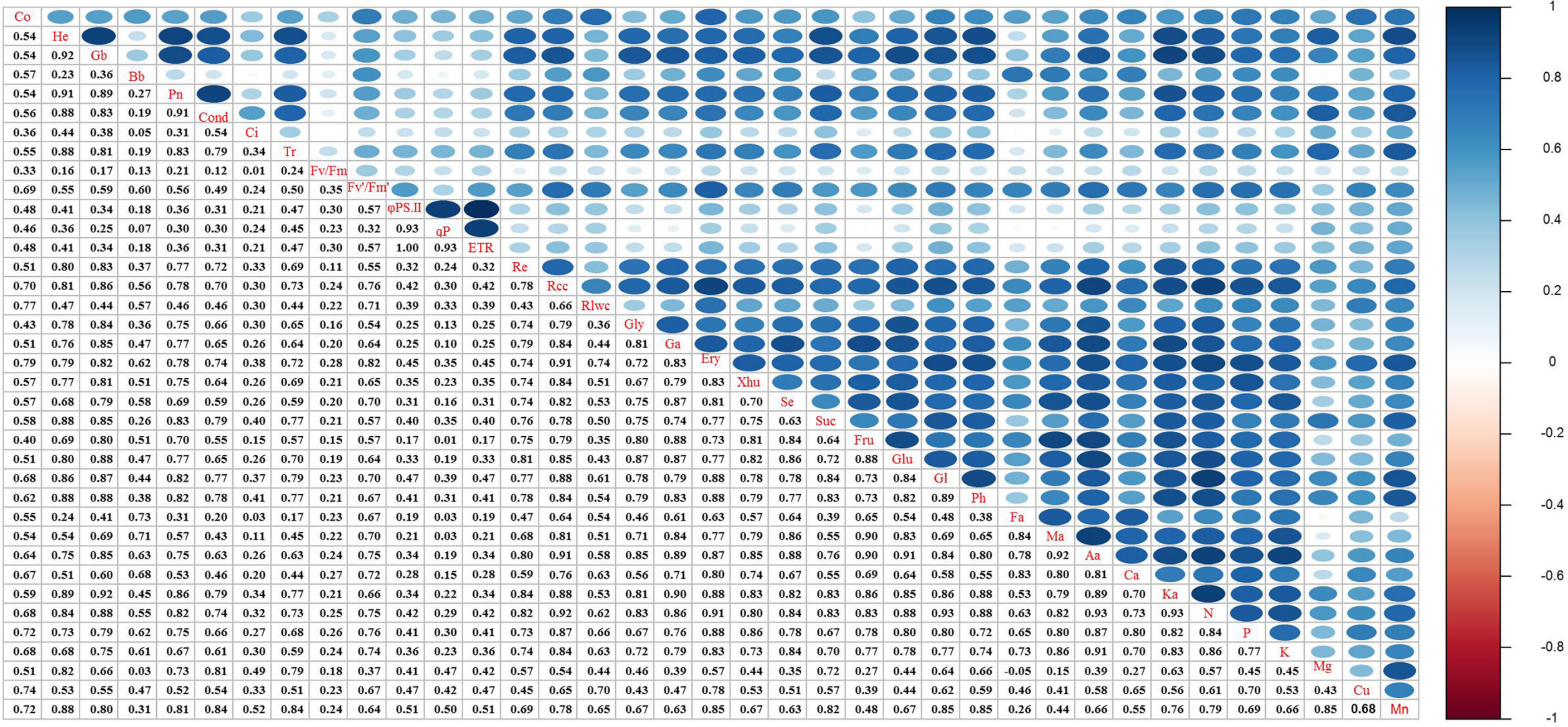
Effects of grazing rest during the spring regreening period on quantitative characteristics of degraded alpine meadow vegetation. Pn, Net photosynthetic rate; Cond, Stomatal conductance; Ci, Intercellular CO_2_ concentration; Tr, Transpiration rate; Fv/Fm, Photochemical quantum efficiency; Fv’/Fm’, Effective photochemical quantum efficiency; φPSII, Actual photochemical quantum efficiency; qP, Photochemical quenching coefficient; ETR, Electron transfer rate; Re, Respiratory rate; Co, Coverage; He, Height; Gb, Ground biomass; Bb, Belowground biomass; Rcc, Relative chlorophyll content; Rlwc, Relative leaf water content; Gly, DL-Glyceraldehyde; Ga, Glyceric acid; Ery, D-Erythrose; Xhu, Xylo-hexos-5-ulose; Se, Sedoheptulose; Suc, Sucrose; Fru, Frucose; Glu, Glucose; Gl, Glutamic acid; Ph, Phytol; Ba, Butanedioic acid; Fa, Fumaric acid; Ma, Malic acid; Aa, Aconitic acid; Ca, Citric acid; Ka, α-Ketoglutaric acid.

In this study, all indicators of degraded alpine grassland vegetation were significantly correlated with each other (*P* < 0.05). The correlation coefficients between aboveground biomass and Pn, height and Pn, aboveground biomass and respiration rate, and height and respiration rate were 0.89, 0.91, 0.83, and 0.80, respectively (**Figure 8**). This indicated that the restoration of degraded meadow vegetation by grazing rest during the spring regreening period was significantly correlated with photosynthesis and respiration. Cond, α-ketoglutaric acid, Tr, sucrose, glutamic acid, phytol, and N were highly correlated with Pn, except vegetation quantitative characteristics. In addition, the correlation coefficient between Fv′/Fm′ and erythrose was 0.82. These indicated that the key factors for increased photosynthesis were the supply of reactants involved in the Calvin cycle and the synthesis of chlorophyll. Meanwhile, glyceric acid, α-ketoglutaric acid, glucose, and aconitic acid were highly correlated with respiration rate, except vegetation quantitative characteristics. This suggests that the increase in respiration was due to an increase in the direct reactant content of the TCA cycle.

## Discussion

### Effects of grazing rest during the spring regreening period on the quantitative characteristics of degraded alpine meadow vegetation

Scholars generally agree that grazing rest can improve the vegetation growth of degraded grasslands (Zimmer et al., 2010; Ash et al., 2011; Selemani et al., 2013; Wu et al., 2017). In our study, grazing rest significantly increased the coverage, height, ground biomass, and belowground biomass of the degraded alpine meadow vegetation. Zhao et al. (2016) obtained a similar result in that grazing exclusion enhances plant height, total cover, aboveground biomass, and belowground biomass in the alpine steppe, alpine meadow, and swamp meadow. Bai et al. (2015); Qin et al. (2021), and Chen et al. (2007) also reported that grazing significantly decreases the aboveground biomass of the Inner Mongolia temperate steppe. Bai et al. (2015) indicated that grazing reduces root production. The most important reasons for the improvement of degraded grassland vegetation during grazing rest are to avoid trampling and feeding by livestock and to change the grassland ecosystem environment (Zhao et al., 2016). In addition, the improvement in vegetation quantitative characteristics gradually stabilized with the increase in rest years, which might be caused by the gradual adaptation to the environment without trampling and feeding by livestock. In order to understand the reason why the grazing rest during the spring regreening period promoted the restoration of the degraded alpine meadow vegetation, the photosynthesis and respiration processes were explored in this study. The correlation analysis showed that the quantitative characteristics of the alpine meadow vegetation were significantly correlated with the parameters of photosynthesis and respiration.

### Effects of grazing rest during the spring regreening period on the photosynthesis of degraded alpine meadow vegetation’s dominant grass *E. nutans* Griseb.

The photosynthesis of grass leaves is the primary determinant of carbohydrate sources for plant growth and development (Ellsworth et al., 2004). Our results show that all photosynthetic functions (Pn, Cond, Tr, and Ci) in *E. nutans* were significantly increased by grazing rest. Several researchers have obtained similar results. They found that overgrazing by livestock dramatically restricts leaf photosynthetic capacity and function (such as Pn, Cond, Tr, and Ci) under field conditions (Chen et al., 2005; Zhao et al., 2009; Zheng et al., 2011; Shen et al., 2013; Ren et al., 2017). Chlorophyll fluorescence parameters are highly sensitive to changes in external environmental conditions and can be used to evaluate the effects of external disturbances on plants (Maxwell and Johnson, 2000). Wang et al. (2022) found that chlorophyll fluorescence shows a good correlation with grassland productivity. Our results show that all chlorophyll fluorescence parameters (Fv/Fm, Fv′/Fm′, φPS II, ETR, and qP) of *E. nutans* were significantly increased by grazing rest. Zhang et al. (2022) obtained similar results in that the regulation of chlorophyll fluorescence is constrained under grazing. In addition, Li et al. (2018) found that Fv/Fm, φPSII, and qP are not significantly changed by grazing for 1 year. This may be caused by different climates, grassland types, and dominant grasses. Weng and Lai (2005) and Wang et al. (2022) found that the chlorophyll fluorescence parameters are decreased by heat and drought tolerance. These results indicate that the increase in chlorophyll fluorescence parameters caused by grazing rest resulted from the elimination of grazing stress. However, there have been no detailed reports on why grazing rest promotes photosynthesis in herbage. By using metabolomic techniques, our study demonstrated that grazing rest significantly promoted the Calvin cycle in herbage. We found that the levels of eight metabolites related to the Calvin cycle significantly increased under grazing rest, and sucrose and erythrose had a higher correlation with related parameters of photosynthesis among the eight enriched metabolites. At present, the relationship between grazing rest and the Calvin cycle has not been reported.

Moreover, the photosynthetic responses in different grazing rest years were compared in this study. We found that all photosynthetic functions and chlorophyll fluorescence parameters significantly increased first and then gradually stabilized. These results might be caused by the similar pattern of changes in the eight metabolites in the Calvin cycle, chlorophyll content, leaf relative water content, and mineral element content. Chlorophyll, a photosynthetic pigment, plays an important role in the absorption and utilization of light energy by green plants. Chlorophyll content is closely related to the level of plant photosynthesis. Many other researchers have reported similar results in that grazing rest induces more chlorophyll than grazing (Thomas, 2012; Ren et al., 2017). Furthermore, we used metabolomics techniques and found that the increased chlorophyll content resulted from the increased levels of glutamic acid and phytol, which are metabolites related to chlorophyll biosynthesis, and the correlation coefficient between glutamic acid and relative chlorophyll content was 0.87. However, this may result from the decrease in plant leaf water and soil water contents after grazing disturbance, leading to some degradation of chlorophyll in forage leaves (Sohrabi et al., 2017). In this study, not only was leaf relative water content absolutely increased by grazing rest but also the N, Mg, and Cu contents in leaves. N and Mg are essential elements for chlorophyll biosynthesis (Rissler et al., 2002; Eckhardt et al., 2004). The Cu supply significantly increases the Chl-a concentration (Biswas et al., 2013). In addition to promoting chlorophyll biosynthesis, mineral elements play important roles in other photosynthetic processes. Nobuyuki et al. (2008) pointed out that N, P, and K deficiencies significantly decrease photosynthesis and RuBP carboxylase–oxygenase activity in rice (*Oryza sativa*) plants. Tanwar et al. (2013) indicated that phosphate application significantly increases photosynthesis and stomatal conductance in bell pepper (*Capsicum annuum*). Mn is indispensable for water splitting during photosynthesis (Eisenhut et al., 2018). Our results show that the N, P, K, Mg, Cu, and Mn contents were significantly increased by grazing rest. Yin and Lu (2013) obtained a similar result; *Leymus chinensis* displays increasing leaf N and P concentrations over time after grazing exclusion. Harrison et al. (2010) stated that grazing may affect photosynthesis as a consequence of changes in leaf water status, nitrogen content, and photosynthetic enzymes. Moreover, the variation trends of mineral elements and leaf relative water content with grazing rest years are consistent with the variation trends of the parameters related to underground biomass and photosynthesis. Therefore, it was reasonable to infer that grazing rest could enhance the grass absorption of mineral elements and water by increasing the underground biomass of herbage, thereby promoting chlorophyll biosynthesis and photosynthesis.

### Effects of grazing rest during the spring regreening period on the respiration of degraded alpine meadow vegetation’s dominant grass *E. nutans* Griseb.

Plant respiration is a basic process of plant physiological metabolism that provides energy for plant life activities (Noguchi and Yoshida, 2008). Respiration and photosynthesis cooperate closely in plant energy metabolism (Archontoulis et al., 2012). Similar to the net photosynthetic rate, the respiration rate of the degraded alpine meadows vegetation’s dominant grass *E. nutans* Griseb. significantly increased by grazing rest during the spring regreening period. There are very few reports on the effects of grazing rest or grazing on plant leaf respiration rate. Shen et al. (2013) indicated that the dark respiration rate of *Gentiana straminea* in un-grazed regimes is higher than that in grazed regimes under ambient conditions. Furthermore, we used metabolomics to determine which metabolic process of respiration responds to grazing rest, and the results show that six key metabolites in the TCA cycle were significantly enriched and that aconitic acid and ketoglutaric acid had a higher correlation with respiration rate among the six enriched metabolites. In addition, a significant increase in respiration factors was also detected owing to grazing rest in this study. Water is necessary for respiration (Galmes et al., 2007). The rate of respiration is lower in P-deficient plants than in P-fertilized plants (Bahar et al., 2018). K enhances the respiration of *Nicotiana tabacum* by increasing the O_2_ uptake (Wakhloo and Ruppenthal, 1989). Copper oxidase is involved in the reduction of oxygen molecules in plants and has significant effects on plant respiration (Patterson, 2013). Mn increases the respiration intensity of plants and regulates redox processes (Hsieh, 2011). This may be because grazing rest could enhance the respiration of the degraded alpine meadow vegetation’s dominant grass *E. nutans* Griseb. 

## Conclusion

Grazing rest during the spring regreening period promoted the ecological restoration of degraded alpine meadows by enhancing the photosynthesis and respiration of the dominant grass *E. nutans* Griseb. Grazing rest enhanced photosynthesis in dominant grass by increasing the metabolites related to the Calvin cycle, chlorophyll content, leaf relative water content, and related mineral element content. Grazing rest also enhanced the respiration of dominant grass by increasing the metabolites related to the TCA cycle, leaf relative water content, and related mineral element content. 

## Data availability statement

The data analyzed in this study is subject to the following licenses/restrictions: according to state policies, requests to access these datasets should be directed to YL, liuying_yanhong@sina.com. 

## Author contributions

The author confirms being the sole contributor of this work and has approved it for publication.

## Funding

This research was supported by the National Natural Science Foundation of China (U21A20241) and the Key Laboratory Project of Qinghai Province, China (2020-ZJ-Y03). 

## Acknowledgments

The author would like to thank Yushou Ma and Shixiong Li for providing the experimental field, and Wenhui Liu for providing the instruments facility. The author is grateful to all editors and reviewers for their valuable suggestions on the manuscript.

## Conflict of interest

The author declares that the research was conducted in the absence of any commercial or financial relationships that could be construed as a potential conflict of interest. 

## Publisher’s note

All claims expressed in this article are solely those of the authors and do not necessarily represent those of their affiliated organizations, or those of the publisher, the editors and the reviewers. Any product that may be evaluated in this article, or claim that may be made by its manufacturer, is not guaranteed or endorsed by the publisher.
